# G-quadruplexes self-assembled from nucleotide monomers as stable prepolymer scaffolds in aqueous environments

**DOI:** 10.1038/s41598-026-38899-5

**Published:** 2026-02-07

**Authors:** Simon H. J. Eiby, Thomas E. Catley, Max C. Gamill, Alice L. B. Pyne, Tue Hassenkam

**Affiliations:** 1 Globe Institute, University of Copenhagen, Copenhagen, 1350 Denmark; 2 School of Chemical, Materials and Biological Engineering, University of Sheffield, Sheffield, UK

**Keywords:** Biochemistry, Biophysics, Biotechnology, Chemical biology, Chemistry, Structural biology

## Abstract

Life is composed of genetic and functional polymers, such as nucleic acids. For life to have emerged, a prebiotic mechanism for assembling these polymers is essential. In a prebiotic environment teeming with diverse organic molecules, selecting, concentrating and bringing together only relevant building blocks poses a significant challenge. G-quadruplexes, a secondary structure of DNA and RNA, are known to self-assemble from nucleotide monomers, creating an ideal preassembly for nucleotide polymerization. We investigate the detailed structure of self-assembled G-quadruplexes using high-resolution atomic force microscopy (AFM) measurements in solution. We show that G-quadruplexes of nucleotide monomers are stable on surfaces in aqueous solution at concentrations orders of magnitude below their solubility limit. When subjected to cycles of evaporation and rehydration at elevated temperatures, the G-quadruplexes partially transform into extended, RNA-like structures, also stable on surfaces in solution, consistent with a polymeric nature. G-quadruplexes self-assembled from nucleotide monomers could have served as persistent prepolymer scaffolds, providing genuine molecular selectivity in prebiotic environments.

**Supplementary Information:**

The online version contains supplementary material available at 10.1038/s41598-026-38899-5.

## Introduction

The emergence of life from prebiotic molecular feedstocks on the early Earth is a remarkable transition, yet far from understood. Multiple hypotheses have been proposed^1–5^ but regardless of the actual pathway that led to the origin of life, a central problem exists: How were the molecular building blocks of life selected, concentrated and brought together to form the genetic and functional polymers required for life to begin in earnest?

Nucleotides are the building blocks of nucleic acids, RNA and DNA, the information bearing molecules of life. Besides carrying genetic information, RNA can also exhibit catalytic properties, which has resulted in the RNA world hypothesis^2,6,7^, where RNA, or a similar polymer, is proposed to have preceded DNA and proteins as a combined genetic and functional molecule in an early life form. In support of the RNA world hypothesis, it has been demonstrated that RNA is capable of replicating and synthesising other RNAs in absence of proteins^8–10^. RNA nucleotides are composed by nucleobases (guanine, cytosine, adenine and uracil), ribose and phosphate, which all potentially were present individually on the prebiotic Earth, either through prebiotic synthesis on Earth^11–14^ or via extraterrestrial sources^15–18^. Despite challenging, potential prebiotic pathways to RNA nucleosides (nucleotides lacking the phosphate group) and nucleotides have been demonstrated^19–26^. However, if nucleotides existed on the prebiotic Earth, they were likely present at relatively low concentrations and coexisted with a wide variety of other molecules.

Nucleotide polymerization through the formation of covalent phosphodiester bonds is endergonic and does not occur spontaneously in aqueous solution. Drying can promote polymerisation and repeated cycles of evaporation and rehydration have been suggested to drive and sustain non-enzymatic polymer formation^5,27^. The early Earth likely featured subaerial land masses with freshwater pools^28,29^. In these pools, nucleotides could accumulate upon drying, polymerize in the dry state, and reorganise in the wet phase. Supporting this, experimental studies have shown that RNA-like polymers can form non-enzymatically by wet/dry cycling, both under alkaline, room temperature to warm conditions using intrinsically activated nucleotides^30,31^ and under acidic and hot conditions using regular non-activated nucleotides^32–34^.However, for nucleotide polymerization to occur in prebiotic environments, a robust prebiotic selection mechanism is required to concentrate these monomers and maintain their close proximity. Environmental factors such as UV radiation^35^ and heat flows through interconnected rock fractures^36^ have been proposed as prebiotic selection mechanisms but lack true molecular specificity. To the best of our knowledge, no prebiotic catalyst, i.e. mineral surface, capable of selecting and binding a specific type of molecule has been identified so far. However, self-assembly processes can induce order in prebiotic environments^37^, and one of the nucleotides, guanosine monophosphate (GMP), holds unique intrinsic self-assembling capabilities.

Highly concentrated guanine-based units, such as GMP, are known to form stable hydrogen-bonded G-quartets, stacking on top of each other in presence of cations (typically K^+^ and Na^+^) forming long rods/wires of G-quadruplexes without the formation of any covalent bonds^38,39^. The G-quadruplex structure has been known for more than 60 years^40,41^ and has since been identified as a very stable naturally occurring secondary structure of RNA/DNA with relevance for replication, transcription and other biological processes^42,43^. Thus, G-quadruplexes represent a structure that can self-assemble both from GMP monomers and in G-rich RNA/DNA polymers, constituting a structural connection between prebiotic molecules and essential biopolymers, i.e. RNA or DNA. It has been suggested that GMP monomers in self-assembled G-quadruplexes are perfectly lined up for the formation of phosphodiester bonds^39,44^. On that basis, it has been hypothesised by Kankia^45^ that prebiotic RNA synthesis occurred on self-assembled G-quadruplexes towards the origin of life, termed the “Quadruplex World”. Supporting this, a recent study has shown that intrinsically activated GMP monomers exhibit a markedly higher propensity to oligomerize (up to 10-mers) than other similarly activated nucleotides, following their self-assembly into G-quadruplexes^31^.

**Fig. 1:**
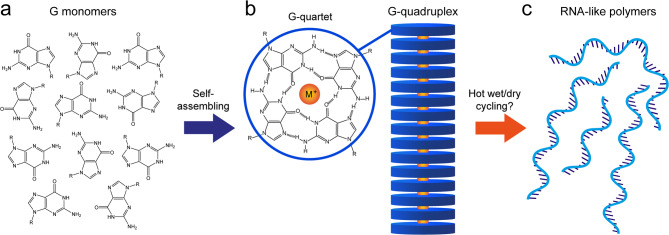
Conceptual depiction of the potential prebiotic formation of RNA-like polymers scaffolded by self-assembled G-quadruplexes. (**a**) Guanine-based monomers, like GMP, self-assemble into G-quadruplexes upon drying (**b**). The G-quadruplexes are composed of stacks of G-quartets, with monovalent cations, typically K^+^, chelated between neighboring G-quartets. A G-quartet is composed of four G monomers linked together by hydrogen bonds in a planar arrangement. (**c**) By subjecting these self-assembled G-quadruplexes to hot (80 °C) wet/dry cycles, phosphodiester bonds may form between G monomers in neighboring G-quartets resulting in RNA-like polymers.

Here we show that G-quadruplexes self-assembled from GMP monomers are stable on mica surfaces in buffered aqueous solution containing K^+^, at total GMP concentrations below 1 µM. Using high-resolution atomic force microscopy (AFM) measurements in solution, we probe the detailed structure of these G-quadruplexes and how they partially dissolve in a destabilising ionic environment. Furthermore, we show that the self-assembled G-quadruplexes of GMP monomers can transform into RNA-like structures when subjected to hot (80 °C) wet/dry cycles. These structures are also stable on the surface in aqueous solution and contract when altering the ionic environment, consistent with a polymeric nature. However, the specific configuration of GMP monomers and type covalent linkages within these RNA-like structures remain unresolved. In accordance with the hypothesis by Kankia^45^ and as depicted in Fig. 1a–c, GMP monomers self-assemble into G-quadruplexes, forming stable prepolymer scaffolds that promote selective concentration and organization for polymerization to occur, potentially driven by hot wet/dry cycling.

## Results

### Self-assembling of G-quadruplexes

First, G-quadruplexes (G4s) were assembled from guanosine monophosphate (GMP) monomers. Solutions of GMP were dried on mica surfaces in a desiccator at room temperature and the surfaces inspected with AFM. A relatively thick (> 10 nm) layer of filamentous structures was observed on the surface before washing (Fig. S1a). After washing, the thick layer had disappeared, and distinct long filaments, measuring from < 100 nm up to more than 1 μm in length and between 1.2 and 1.8 nm in height, were observed on the otherwise clean mica surface (Fig. S1b). These filamentous structures are interpreted as self-assembled G4s. Firstly, the morphology and dimensions match previous AFM analyses, conducted in air, of G4s formed by self-organized GMP monomers^38^ and G-rich oligonucleotides^46^. Secondly, GMP monomers (in their salt forms) are known to be capable of self-assembling into G4s when reaching a certain critical concentration in solution^39,44^. Lastly, the starting GMP solutions were prepared in ultrapure MQ water, filtered using low molecular weight cut-off membranes and used shortly hereafter. Thus, the risk of contaminant has been minimized.

**Fig. 2:**
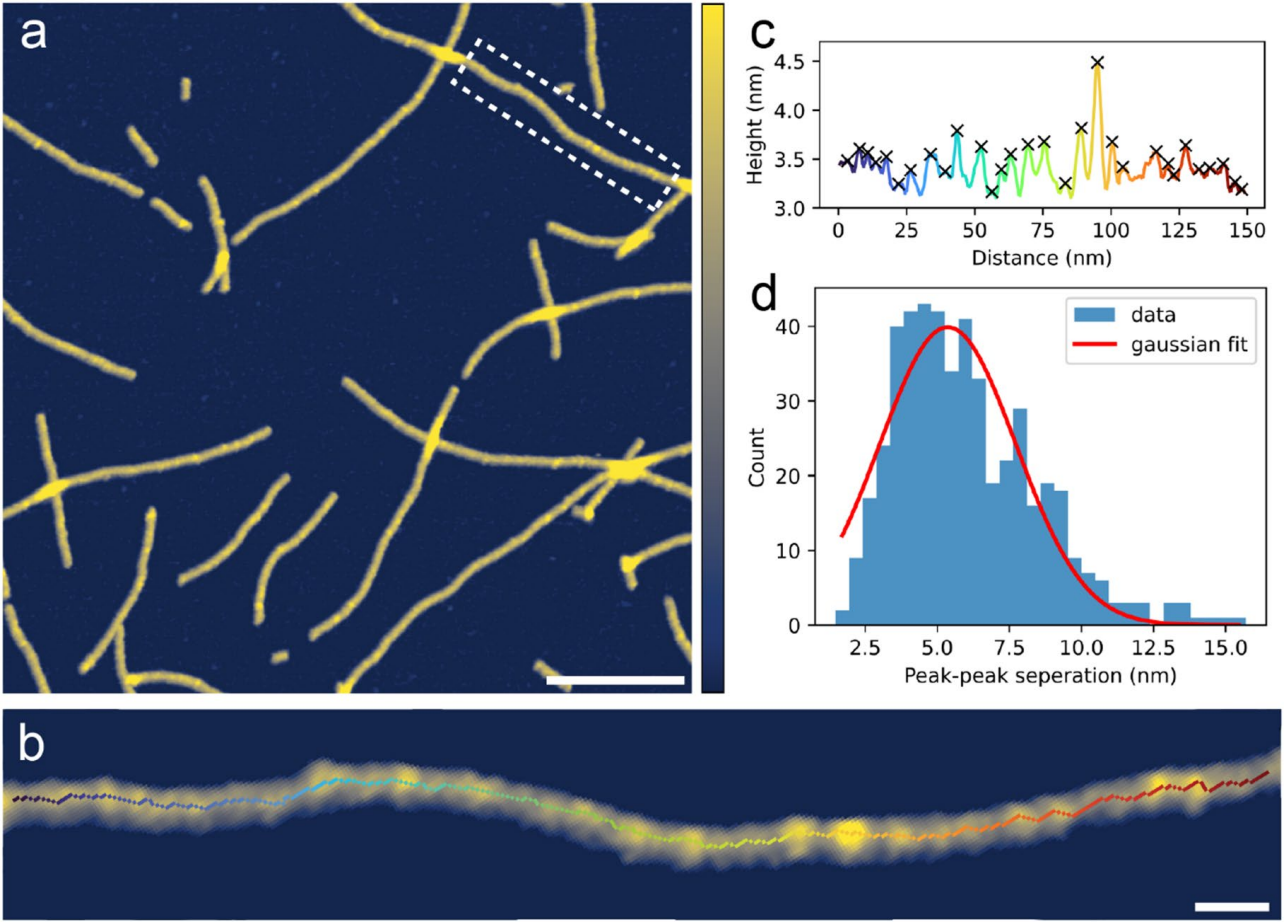
PeakForce Tapping^®^ AFM images of GMP solution dried on mica in a desiccator at room temperature, followed by thorough washing using MQ water. Imaging conducted in 100 mM KCl + 20 mM HEPES pH 7.4 aqueous solution. (**a**) Representative overview image of the G-quadruplexes. Scale bar is 100 nm and color z-scale is 0–4 nm. (**b**) Close-up image of a single G-quadruplex segment including skeletonised trace. Scale bar is 10 nm and color z-scale is 2–4 nm. (**c**) Corresponding height profile along skeletonised trace with identified peaks marked with crosses. (**d**) Histogram of peak-peak separations in height profiles from multiple G4s in several images (*N* = 33 molecules).

The self-assembled G4s were imaged using AFM in a buffered KCl solution and appeared as long filamentous structures which were stable on the surface at least for the measurement period (Figs. 2a and S2a, b). Because the surfaces with self-assembled G4s were thoroughly washed prior to AFM measurements, the effective concentration of GMP on the surface relative to the volume of the imaging solution was low (< 1µM, based on surface coverage and assuming homogenous G4 distribution across the mica substrate). Thus, even with no covalent linkages between the GMP monomers and relatively low concentration, the self-assembled G4 structure exhibited stability in an aqueous environment over hours. Besides the G4s, the mica surface appeared clean, free from any other particles. The G4s varied in length, with the shortest measuring around 10 nm and the longest more than 1 μm. In regions rich in G4s, the structures frequently overlapped but in general, the G4s exhibited the same overall morphology as when imaged in air (Fig. S1b). Figure 2b shows a close-up image of a G4 segment, and Fig. 2c shows a corresponding height profile depicting the typical topographical variations observed along the structures. Peak finding (by prominence) was conducted on the height profiles of multiple G4s from several images (*N* = 33 molecules) and peak-peak separations were calculated and plotted together in a histogram Fig. 2d. A gaussian fit was applied to the peak-peak separation distribution and the mean was 5.4 ± 2.4 nm. If 3.4 Å is the distance between two neighbouring G-quartets in a G4, 5.4 ± 2.4 nm correspond to 16 ± 7 stacked G-quartets. This structural repetition suggests that the G4s formed through the stacking of discrete structural units, each comprising several stacked G-quartets, rather than by continuous extension of one G-quartet at a time. Notably, the size of these structural units is comparable to one full helical turn of a previously reported self-assembled G4 structure^39^. As a measure for height variation along the G4 structures, the RMS roughness for all the height profiles was calculated and averaged to 0.21 ± 0.07 nm (*N* = 33). In general, the G4s displayed a range of different topographical variations, as indicated by the relatively large standard deviation on the peak-peak separation (2.4 nm) and RMS roughness. Some segments displayed fine periodic variations, while others displayed more bulky irregular variations (more examples in Fig. S3). Thus, the self-assembly process upon the extended drying at room temperature promotes some degree of disorder in the structures.

The mean height of the self-assembled G4s was calculated to be 3.4 ± 0.2 nm (*N* = 33), by averaging the height values along the central axes of multiple G4s (Fig. 2b, c). G4s formed by self-assembling GMP monomers have not previously been examined by AFM in aqueous ionic solutions but the height reported here corresponds well to previous AFM analyses of G4s formed by G-rich oligomers in comparable solutions, where a height of 3.0 ± 0.3 nm was reported^47^.

To confirm that the G4s were not present in the starting solutions but formed in the extended drying process, GMP solutions were incubated on mica surfaces for 5 min, followed immediately by gentle rinsing and either (1) drying for AFM measurements in air or (2) addition of a KCl solution for liquid AFM measurements. Figure S4a, b shows AFM images of the mica surfaces after GMP incubation, and no filamentous structures were observed. The GMP solution was also incubated on mica under N_2_ for 1 h, followed by gentle rinsing and drying for AFM measurements in air (Fig. S5). Significantly more material was present on the surface after 1 h incubation under N_2_, compared to the 5 min incubations, and few short rod-like structures were observed (indicated by arrows), implying that the drying stages are essential for the formation of G4 structures.

### Stability of self-assembled G-quadruplexes

**Fig. 3:**
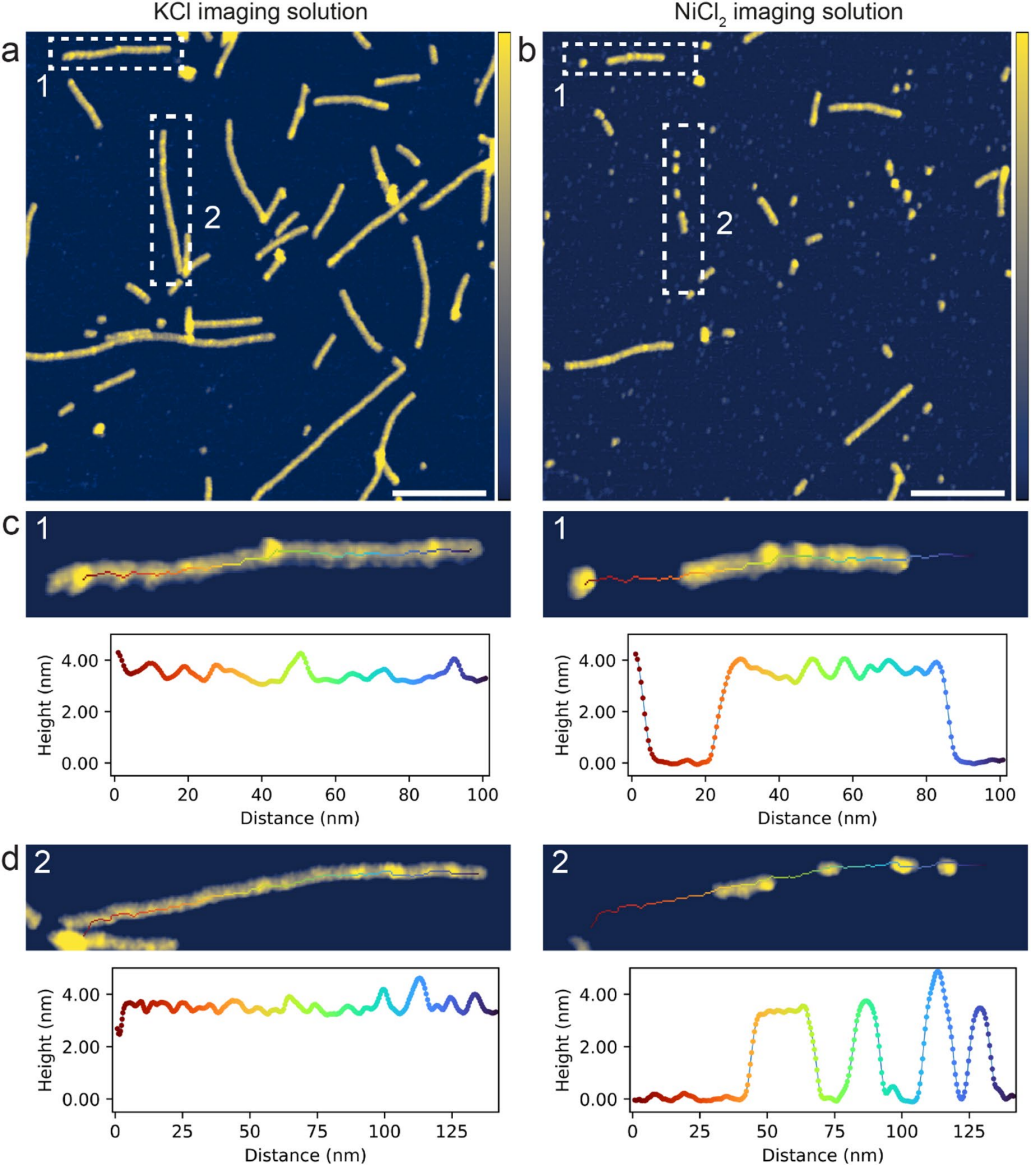
PeakForce Tapping^®^ AFM images of GMP solution dried on mica in a desiccator at room temperature, followed by thorough washing using MQ water. (**a**) Imaged in 100 mM KCl + 20 mM HEPES pH 7.4, scale bar is 100 nm and color z-scale is 0–4 nm. (**b**) The same location as in ‘a’ but after a complete exchange of the imaging solution to 3 mM NiCl_2_ + 20 mM HEPES pH 7.4, scale bar is 100 nm and color z-scale is 0–4 nm. (**c**–**d**) Close-up images and corresponding height profiles of two G-quadruplex segments (labelled ‘1’ and ‘2’) before and after exchanging the imaging solution from containing KCl to NiCl_2_, color z-scale is 2–4 nm.

The stability and nature of the self-assembled G4s was examined by exchanging the imaging solution to a buffered aqueous solution containing NiCl_2_ instead of KCl. As the imaging solutions are buffered at neutral pH, both the mica surface and the nucleotides (phosphate groups) are negatively charged, leading to electrostatic repulsion. Cations are used to screen and bridge opposing charges, promoting adsorption. Monovalent K^+^ is known as the optimal cation to support the G4 structure^48^ and screen the negatively charged mica surface, enabling adsorption of stable G4 assemblies, but not individual GMP monomers^49^. In contrast, divalent Ni^2+^ can bridge the repulsive charges and exhibits much stronger binding and coordination to nucleotides in general, and is thus commonly used to effectively immobilize nucleic acids on mica surfaces^49,50^. Figure 3a shows an AFM image of the self-assembled G4s imaged in KCl and Fig. 3b shows an AFM image at the same location but imaged in NiCl_2_. Figure 3c shows dissolution of the G4 filament ‘1’ when exchanging the imaging solution. In the KCl buffer, the G4 presented as a long, continuous structure, and in the NiCl_2_ buffer, as a fragment with a gap to a shorter segment, which constitutes approximately half of the original G4 length. A second G4 segment ‘2’ was also imaged in the KCl and NiCl_2_ solutions (Fig. 3d), and experienced greater dissolution with only four short fragments remaining after the imaging solution was exchanged. For the G4 segments that remained on the surface in the NiCl_2_ solution, the height profiles before and after exchanging the imaging solution did not match in general. Thus, the G4 structures can, in addition to disassemble completely, change conformation when varying the ionic environment.

In general, after exchanging the imaging solution to NiCl_2_, the majority of G4s were heavily fragmented or removed entirely, supporting that the G4s had been destabilised when exchanging K^+^ with Ni^2+^ in the aqueous environment. Previous AFM investigations have shown that G4s formed in G-rich DNA disassembled in absence of salts (KCl) in the imaging solution^51^. When imaging in NiCl_2_, we would expect nucleic acid polymers to be strongly immobilised to the mica surface^49,50^. The extensive fragmentation in NiCl_2_ supports that the G4s are indeed composed by self-assembled GMP monomers and are not covalently linked GMPs or a result of any type of contamination. Some G4 fragments were preserved on the surface when imaging in NiCl_2_, at least within an hour, suggesting that self-assembled G4s were to some extent resistant to the destabilising ionic environment. However, over time, the degree of fragmentation increased as illustrated in the short time series of AFM images shown in Fig. S6a–c. Some structures might be more resilient to disassembly than others and could become dominant on surfaces in a natural aqueous environment over time.

### RNA-like structures produced by hot wet/dry cycling

**Fig. 4:**
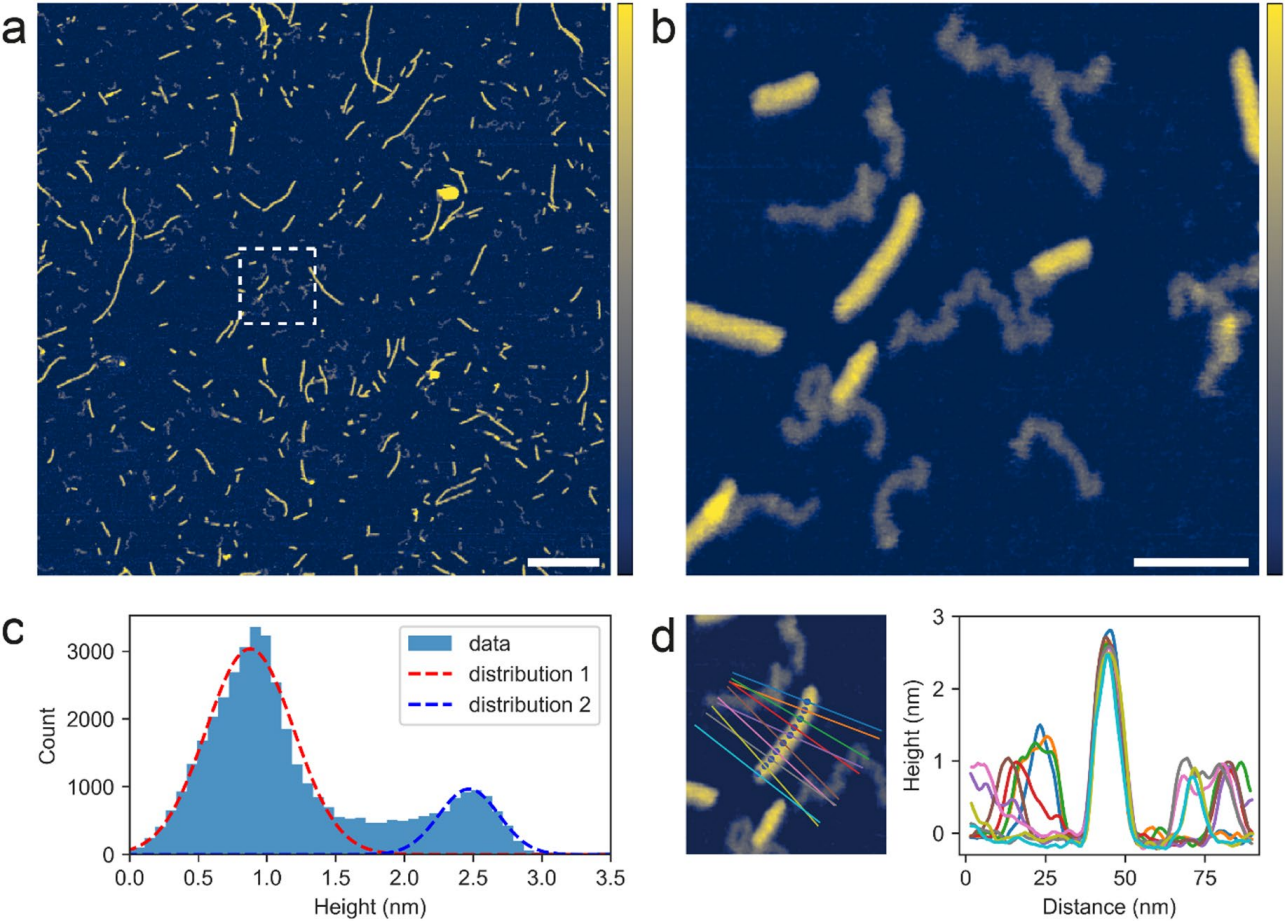
PeakForce Tapping^®^ AFM images of the products after hot (80 °C) wet/dry cycles of the self-assembled G-quadruplexes. Imaging conducted in 100 mM KCl + 20 mM HEPES pH 7.4 aqueous solution. (**a**) Overview AFM image of the products after wet/dry cycling. Dashed box shows area for close-up inspection. Scale bar is 250 nm and color z-scale is 0–3 nm. (**b**) Close-up AFM image of the products after wet/dry cycling. Scale bar is 50 nm and color z-scale is 0–3 nm. (**c**) Height distribution of the pixels (background pixels subtracted) in ‘b’ and gaussian fits of the distributions. (**d**) A series of orthogonal height profiles along a G-quadruplex.

The self-assembled G4s (visualized in Figs. 2a and S2a, b), were subjected to hot (80 °C) wet/dry cycles (3 cycles of 30 min each). Detailed structural analyses of the products after the wet/dry cycles were conducted by AFM imaging in buffered KCl solutions. An overview AFM image is shown in Fig. 4a, revealing numerous filamentous G4-like structures of various lengths, generally spanning 20–1000 nm but typically < 200 nm long, exhibiting comparable dimensions and morphologies as the self-assembled G4s (Figs. 2a and S2a, b). In addition to the G4s, Fig. 4a reveals another type of structure, namely thin coiled polymeric structures dispersed all over the surface and mixed in with the G4s, which is emphasized in the close-up image shown in Fig. 4b. Both the G4s and the thin polymeric structures were stable on the surface in aqueous solution at least for the measurement period (hours). The thin polymeric structures, typically spanning 50–100 nm in length, appeared both independently on the surface and as tail-like extensions of the G4s in ~ 50% of the cases. This suggests that the G4s and the thin polymeric structures are structurally connected and both composed of GMP. The G4 and polymeric structures present in Fig. 4b were masked (Fig. S7a-d) and a height distribution of only the pixels under the mask is shown in Fig. 4c, visualizing the presence of the two distinct distributions of heights. Gaussian fits were applied to the height distributions resulting in: (1) a peak at 0.9 ± 0.3 nm and (2) a peak at 2.5 ± 0.2 nm. A series of height profiles orthogonal to a G4 are shown in Fig. 4d, confirming that the 0.9 nm peak represents the thin coiled polymeric structures, and the 2.5 nm peak represents the G4s. As a control, a clean mica surface was subjected to identical hot (80 °C) wet/dry cycles, rehydrated with the same MQ water, and analysed with AFM. A representative AFM image is shown in Fig. S8, with no structures observed. We therefore conclude that the thin coiled polymeric structures originated from the self-assembled G4s and are thus made by the same material, GMP.

To probe how the G4 structures changed in response to wet/dry cycles, height profiles were taken along the G4 traces and peak-peak separations calculated (Fig. S9a). The mean peak-peak separation along height profiles of G4s after wet/dry cycles was found to be 5.1 ± 2.4 nm (*N* = 15), thus comparable to before wet/dry cycles (5.4 ± 2.4 nm). However, the RMS roughness of the height profiles after wet/dry cycles was found to be 0.07 ± 0.02 nm, substantially lower than before wet/dry cycles (0.21 ± 0.07). The distribution of RMS roughness from multiple G4s before and after wet/dry cycles is shown in Fig. S9b with a clear shift to lower values observed after wet/dry cycles. This supports the observations from the AFM images (Figs. 2b and 4b), in that the G4s appeared smoother after wet/dry cycles, compared to before, exhibiting fewer bulky structural units. Using the same approach as before wet/dry cycles, the mean height of G4s after wet/dry cycling was calculated to be 2.6 ± 0.1 nm (*N* = 15). Comparing to the mean height of the self-assembled G4s before wet/dry cycling (3.4 ± 0.2 nm), the mean height is significantly lower after wet/dry cycling. This fits well with the decrease in RMS roughness, implying that the G4 structure became smoother and more ordered, as a result of the hot wet/dry cycles. This may improve alignment between the GMP monomers to enable the formation of covalent linkages between GMP monomers.

We have shown that self-assembled G4s subjected to hot wet/dry cycles produce thin coiled polymeric structures that are stable on mica surfaces in buffered KCl solution for hours. If these thin structures were non-covalently bonded aggregates of GMP monomers formed during the wet/dry cycles, we would not expect them to be immobilised on the negatively charged mica surface in monovalent cation solutions but readily dissolve^49^. Thus, the coiled polymeric structures most likely contain covalently bonded GMP monomers. The morphology and dimensions of the thin coiled polymeric structures match previous AFM observations of single-stranded RNA standards^52,53^, and their length (typically 50–100 nm) corresponds to 74–148 nucleotide long single-stranded RNA^54^. Albeit there is evidence for a polymeric nature resembling single-stranded RNA, the exact configuration of GMP monomers and covalent linkages, e.g. 3′-5′ or 2′-5′ phosphodiester bonds, within these coiled polymeric structures cannot be confirmed from the results presented here, and they are therefore referred to as RNA-like structures.

### Stability of RNA-like structures

**Fig. 5:**
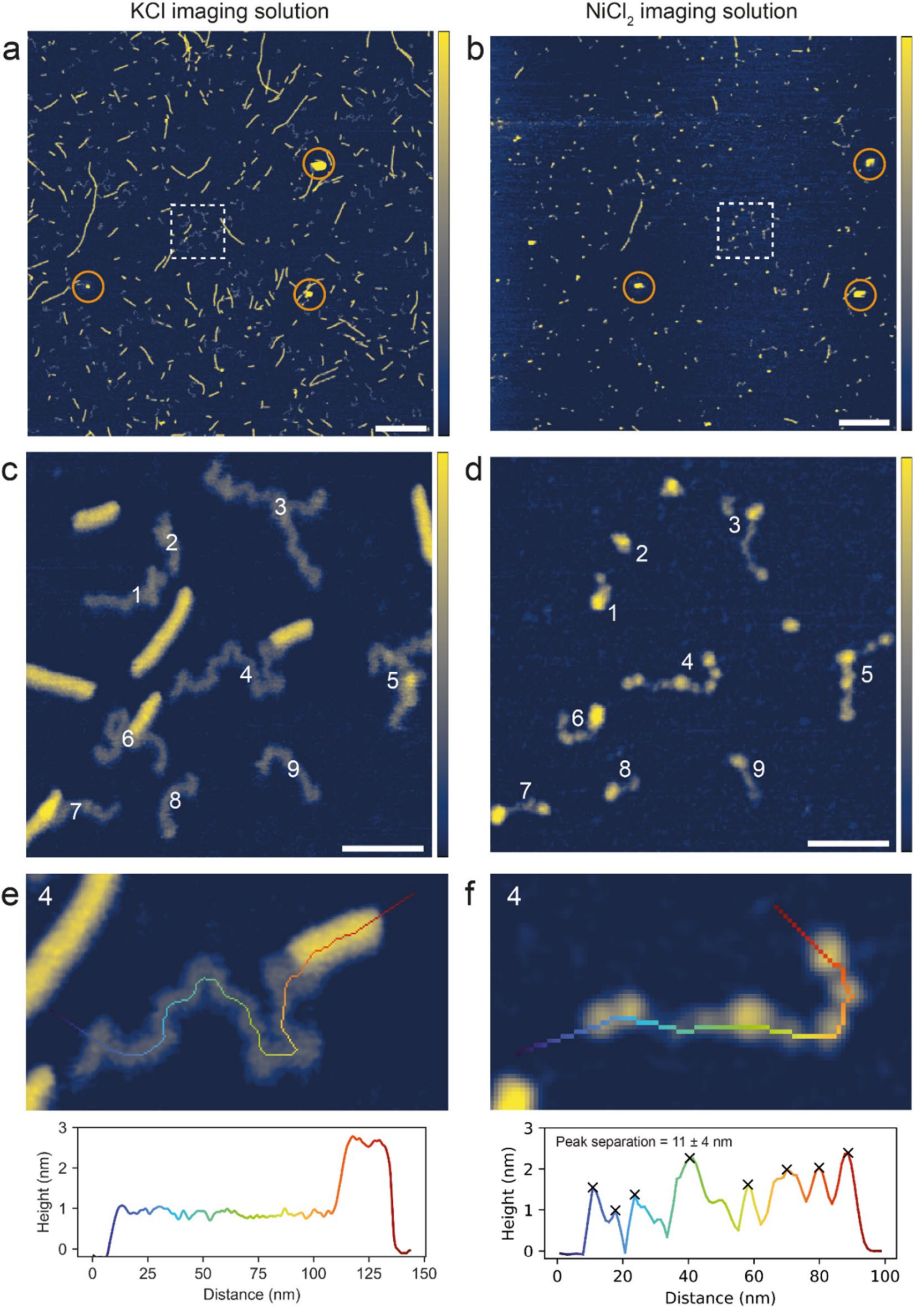
PeakForce Tapping^®^ AFM images of the products after hot (80 °C) wet/dry cycles of the self-assembled G-quadruplexes. Color z-scale is 0–3 nm for all images. (**a**, **b**) Overview images, scale bars are 250 nm, (**a**) imaged in 100 mM KCl + 20 mM HEPES pH 7.4 and (**b**) same location as in ‘a’ but imaged in 3 mM NiCl_2_ + 20 mM HEPES pH 7.4. (**c**, **d**) Close-up AFM images corresponding to dashed boxes in ‘a’ and ‘b’. RNA-like structures are numbered (1–9). Scale bars are 50. (**c**) Imaged in 100 mM KCl + 20 mM HEPES pH 7.4 and (**d**) same location as in ‘c’ but imaged in 3 mM NiCl_2_ + 20 mM HEPES pH 7.4. (**e**, **f**) Close-up AFM images of RNA-like polymer ‘4’ with extended traces shown and corresponding height profiles, (**e**) imaged in 100 mM KCl + 20 mM HEPES pH 7.4 and (**f**) imaged in 3 mM NiCl_2_ + 20 mM HEPES pH 7.4. Identified peaks marked and mean peak-peak separation calculated.

The stability and nature of the G4s and the RNA-like structures formed after wet/dry cycles was examined as before by exchanging the buffered aqueous solution containing KCl, to one containing NiCl_2_ (Fig. 5a, b). In the NiCl_2_ containing buffer (Fig. 5b), the majority of the higher filamentous G4s had disappeared when compared to the same area imaged in the KCl containing buffer (Fig. 5a). This behaviour is comparable to that observed before wet/dry cycles (Fig. 3a, b) and is a result of the ionic environment destabilising the G4 structure. In contrast, many of the thin RNA-like structures were still observed in NiCl_2_, however in a slightly altered form. Close-up AFM images of the same area imaged in KCl and NiCl_2_ are shown in Fig. 5c, d. The individual RNA-like structures are numbered (1–9), enabling a direct comparison when imaging in KCl vs. NiCl_2_. When exchanging the imaging solution from KCl to NiCl_2_, the once uniform in height, RNA-like structures now appeared contracted and as containing dense beads. Height profiles along the trace of the RNA-like structure ‘4’ imaged in KCl (Fig. 5e) and NiCl_2_ (Fig. 5f) show large height variations (between 0.2 and 2.2 nm) when imaged in NiCl_2_, compared to a uniform height of the structure when imaged in KCl. The dense beads observed for the RNA-like structures imaged in NiCl_2_ were typically ~ 2 nm high and the separation between them was 11 ± 4 nm. By exchanging the monovalent K^+^ with divalent Ni^2+^ in the imaging solution, we have shown that the RNA-like structures persisted but changed appearance to a more contracted and denser structure. It has previously been visualized with AFM^55^, that single-stranded RNA is folding (obtain tertiary structure) in the presence of Mg^2+^, whereas the unfolded secondary structure is revealed in absence of divalent cations, which agrees with uniform in height RNA-like structures imaged in KCl. Ni^2+^, although a divalent cation, is slightly different from Mg^2+^ in that it binds and coordinates stronger to RNA, promoting compaction, agreeing with the contracted and denser RNA-like structures imaged in NiCl_2_. This supports that the RNA-like structures contain covalently bonded GMP monomers.

## Discussion

Regardless of the specific chemical pathways involved in the emergence of life, the transition from building blocks of life, such as nucleotides, to genetic and functional polymers, like nucleic acids, is inevitable. Without efficient prebiotic selection mechanisms and stable prepolymer scaffolds, the immense chemical diversity in prebiotic environments might hinder life from emerging. As reasoned previously^45^, a prebiotic environment of randomly polymerized RNA polymers, mixed in with numerous other types of organic molecules, faces significant challenges to foster the emergence of life. The vast number of possible RNA sequences and the relatively small amounts of RNA compared to other molecules dilute any potentially advantageous sequences and structures heavily. In contrast, RNA polymers scaffolded by G4s are homopolymers (poly-G) of varying lengths, reducing the diversity drastically, and constitute a consistent local supply of a specific type of RNA.

In this study, we have shown that G4s self-assembled from GMP monomers are stable on mica surfaces in aqueous solutions containing K^+^, a prebiotically relevant constituent^5^. We have used pure reagents and buffered AFM imaging solutions, which do not resemble messy prebiotic environments, but provide a controlled model system to probe detailed structures and stability under different ionic aqueous conditions. Once formed on the surface, the G4s persist in aqueous environments with very low total GMP concentrations (< 1µM), suggesting that they can accumulate over longer time scales, as long as GMP monomers are supplied. In addition, we show that G4s can also self-assemble when drying dilute solutions of nucleotide mixtures, containing all four canonical nucleotides (Fig. S10a, b), supporting the preferential formation of G4s in more complex mixtures.

The G4s formed in this study were assembled using GMP monomers. In a prebiotic context, GMP is a relatively complex molecule composed of phosphate, ribose and the nucleobase, guanine. However, only guanine is directly involved in the hydrogen-bonding of the G-quartet and the stacking into G4s. In other words, the phosphate-ribose part might not be required for the formation of G4s. To test this, we dried up dilute guanine solutions on mica surfaces and inspected the surfaces with AFM (Fig. S11a, b). Filamentous structures, with morphologies and dimensions matching G4s formed by GMP monomers (Fig. S1b), were abundant, supporting that guanine can in fact self-assemble into G4s in absence of phosphate-ribose. Thus, in addition to GMP, various other guanine-based units can potentially self-assemble into G4s scaffolding the formation of nucleic acids with a wide range of different backbones.

Finally, we have shown that self-assembled G4s transform into extended RNA-like structures when subjected to wet/dry cycling at elevated temperatures. We faced substantial challenges in confirming the identity of these structures using complementary analytical chemistry techniques (methodological details in Supplementary Information). Given the evidence from high-resolution AFM measurements in solution indicating that the RNA-like structures contain covalently bonded GMP monomers, we attribute this lack of detection to the large gap between single molecule and bulk levels of detection. We propose the following factors may contribute to the challenges in identification of the RNA-like structures: low polymer concentration, as polymers are formed from small amounts of GMP monomers and are found dispersed in small surface regions where droplet evaporation occurs; strong adsorption of polymers to the mica substrates after hot wet/dry cycling which may render the polymers less accessible to solution-phase extraction; pronounced heterogeneity in length and exact chemical composition of the products due to uncontrolled, non-catalysed condensation; and the inherent instability of RNA-like polymers in aqueous solution which could affect sample recovery.

In conclusion, the unique structure of the self-assembled G4s provides a stable scaffold that facilitates the selective organization and concentration of GMP monomers into long prepolymer assemblies on surfaces in aqueous environments. G4 self-assembly could have acted as a primitive and persistent selection mechanism in prebiotic environments.

## Methods

### Sample preparation

10 mM guanosine 5’-monophopshate (GMP) solutions were prepared by dissolving the free acid form of GMP (MP biochemicals) into fresh ultrapure Milli-Q (MQ) water (18,2 MΩ·cm resistivity and equipped with a dual wavelength UV lamp for sterilization). The GMP solutions (pH ~ 3) were filtered using Pierce concentrators 3 K MWCO (Thermo Scientific), stored in aliquots in sterile LoBind Eppendorf tubes and used for experiments shortly after filtering to minimize the risk of contamination.

The mica substrates were prepared by fixing 5–10 mm (diameter) muscovite mica discs (SPI Supplies V-4 grade or Agar Scientific) on 15 mm (diameter) steel pucks using 2-part epoxy. This way, substrates could be handled with tweezers without risking contaminating the sample. The mica was always freshly cleaved using adhesive tape prior to each experiment.

A desiccator with activated silica gel was used to dry the GMP solutions at room temperature. Mica substrates were place in the desiccator and 10 ul of 10 mM GMP solution was added to the centre of each mica using sterile pipette tips. The GMP solutions were allowed to dry for at least 24 h and were covered while drying. After drying, the samples were washed thoroughly with 3 × 1 ml MQ water using sterile pipette tips, followed by drying using ultraclean filtered nitrogen gas. Samples were stored for AFM analyses, which were conducted within a few days.

Hot wet/dry cycles were conducted using a laboratory hotplate (Corning), which was preheated to give 80 °C on the sample surface (actual surface temperature verified with infrared thermometer). The samples were placed on the hotplate and 3 cycles of 30 min each were conducted, starting each cycle (except the first) with rehydrating the sample using 10 droplets of 1 µl MQ water using sterile pipette tips. Samples were covered during experiments to avoid contamination from the air. The sample surfaces appeared dry after the first few minutes of each cycle, leaving them in the dry state for most of the cycles. After wet/dry cycles, the samples were taken off the hotplate (no washing performed) and stored for AFM analyses, conducted within a few days.

### AFM imaging

AFM measurements in air were conducted using a Cypher from Asylum/Oxford Instruments equipped with OPUS 240AC-NA probes with a spring constant around 2 N/m and resonance frequency around 70 kHz. The Igor Pro software was used for image acquisition. Images were 512 × 512 pixels and acquired in tapping mode at ambient condition and 1 Hz per line.

PeakForce Tapping^®^ AFM in liquid was conducted using a Mulitmode 8 (Bruker) and a Dimension FastScan (Bruker) equipped with PeakForce-HIRS-F-B (nominal resonance frequency of 100 kHz and spring constant of 0.12 N/m) or FastScan-D (nominal resonance frequency of 110 kHz and spring constant of 0.25 N/m) probes. Samples were imaged in 10–20 ul freshly filtered 100 mM KCl + 20 mM HEPES pH 7.4 or 3 mM NiCl_2_ + 20 mM HEPES pH 7.4 aqueous solutions. The samples were left in the imaging solutions for 15 min before imaging. Images were acquired in PeakForce Tapping QNM mode at a fixed off-resonance frequency (2–8 kHz), minimal force (peak force) and at 1–2 Hz per line. Detailed images were acquired with a ≥ 2px/nm resolution. The imaging solution was exchanged by the following procedure: (1) a snapshot of the optical image of the probe on the mica surface was acquired as reference, (2) the probe was withdrawn and lifted out of the imaging solution, (3) existing imaging solution was removed using sterile pipette tips, (4) new imaging solution was added (3 washes of addition and removal of new imaging solution were conducted to ensure complete exchange), and (5) the probe was immersed in the new imaging solution and engaged on the surface at the same location (recognisable features on the surface, such as particles or mica defects were used for exact reposition).

### Data processing and analysis

AFM data processing and analysis was carried out using the freely available, open-source software TopoStats^56,57^. At first, all raw AFM images were batch processed, including filtering (row alignments and tilt removal) and grain identification (by thresholds) and tracing (skeletonisation). Height profiles were generated from the skeletonised grain traces, which beforehand had been dilated to 3 pixels wide traces and smoothened slightly by a rolling average (over 3 pixels). Peaks in the height profiles were identified by prominence and all peak-peak separations were calculated and gathered from multiple AFM images. Root Mean Square (RMS) roughness, the square root of the average squared deviations from the mean height, was calculated for each height profile. The mean height of G-quadruplexes was calculated by averaging all height values along the height profiles of multiple molecules. Thus, the mean height corresponds to the peak height along the structural traces and is not an average height of all pixel values constituting the G-quadruplex grains. This would have resulted in a lower mean due to the slopes around the molecules, which is highly affected by the AFM tip sharpness and condition.

## Supplementary Information

Below is the link to the electronic supplementary material.

Supplementary Material 1

## Data Availability

AFM data, including raw and processed image files are available via Code Ocean^58^ (10.24433/CO.4460790.v1).

## Code availability

The code used to process the AFM images is publicly available on Github via TopoStats (https://github.com/AFM-SPM/TopoStats) or through the Python Package Index (PyPI) (https://pypi.org/project/topostats/). Python notebooks used to extract and present the processed data (.*topostats* files) are available via Code Ocean^58^ (10.24433/CO.4460790.v1).
