# Supplementary material for: G-quadruplexes self-assembled from nucleotide monomers as stable prepolymer scaffolds in aqueous environments
Source: Sci Rep. 2026 Feb 7;16:7644. doi: 10.1038/s41598-026-38899-5 (PMC12936167; doi:10.1038/s41598-026-38899-5)
Supplement: Supplementary file 1 — Supplementary Material 1 [file 41598_2026_38899_MOESM1_ESM.docx]

**Supplementary Information**

**G-Quadruplexes Self-Assembled from Nucleotide Monomers as Stable Prepolymer Scaffolds in Aqueous Environments**

*Simon H. J. Eiby^1^*, Thomas E. Catley^2^, Max C. Gamill^2^, Alice L. B. Pyne^2^, Tue Hassenkam^1^*

*^1^Globe Institute, University of Copenhagen, DK-1350 Copenhagen, Denmark*

*^2^School of Chemical, Materials and Biological Engineering, University of Sheffield, Sheffield, UK*

**Corresponding author: Simon H. J. Eiby,* [*simon.eiby@sund.ku.dk*](mailto:simon.eiby@sund.ku.dk)

**Supplementary figures**


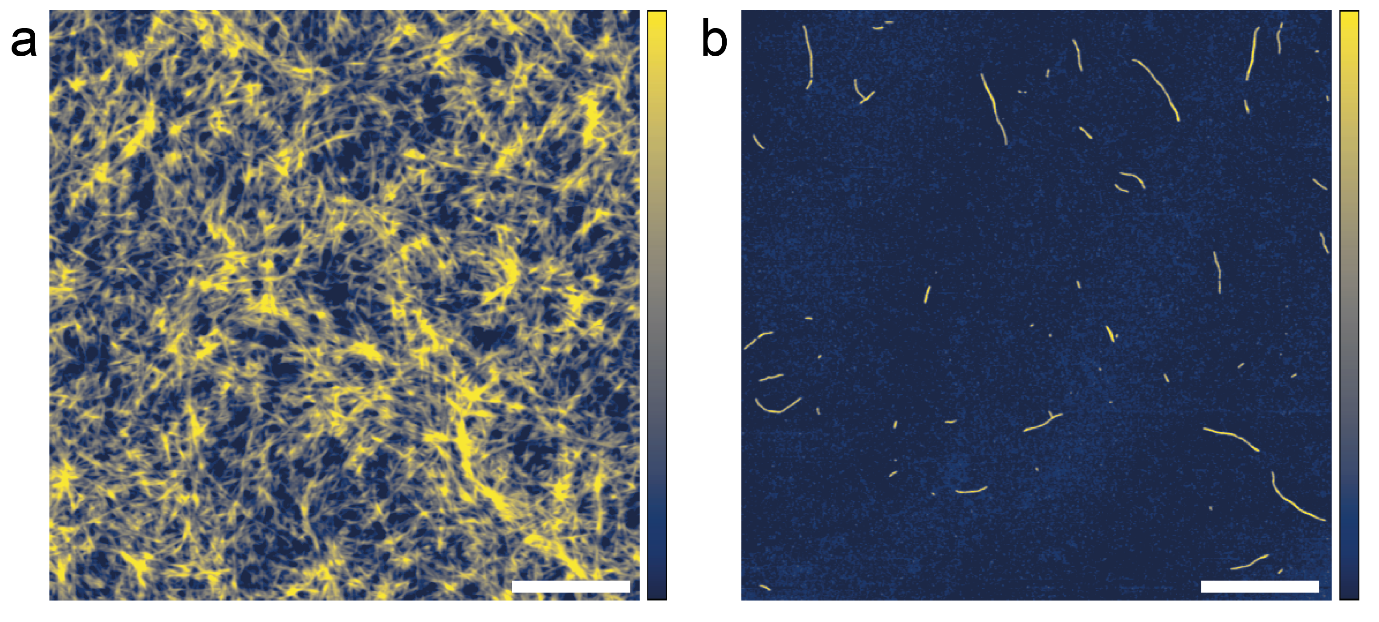


**Figure S1** Representative AFM images of GMP solution dried on mica in a desiccator at room temperature, (a) before washing, color z-scale is 12 nm, and (b) after thorough washing with 3x1 ml MQ water and immediate N_2_ drying, color z-scale is 1.5 nm. AFM conducted in air and the scale bar is 1 µm for both images.


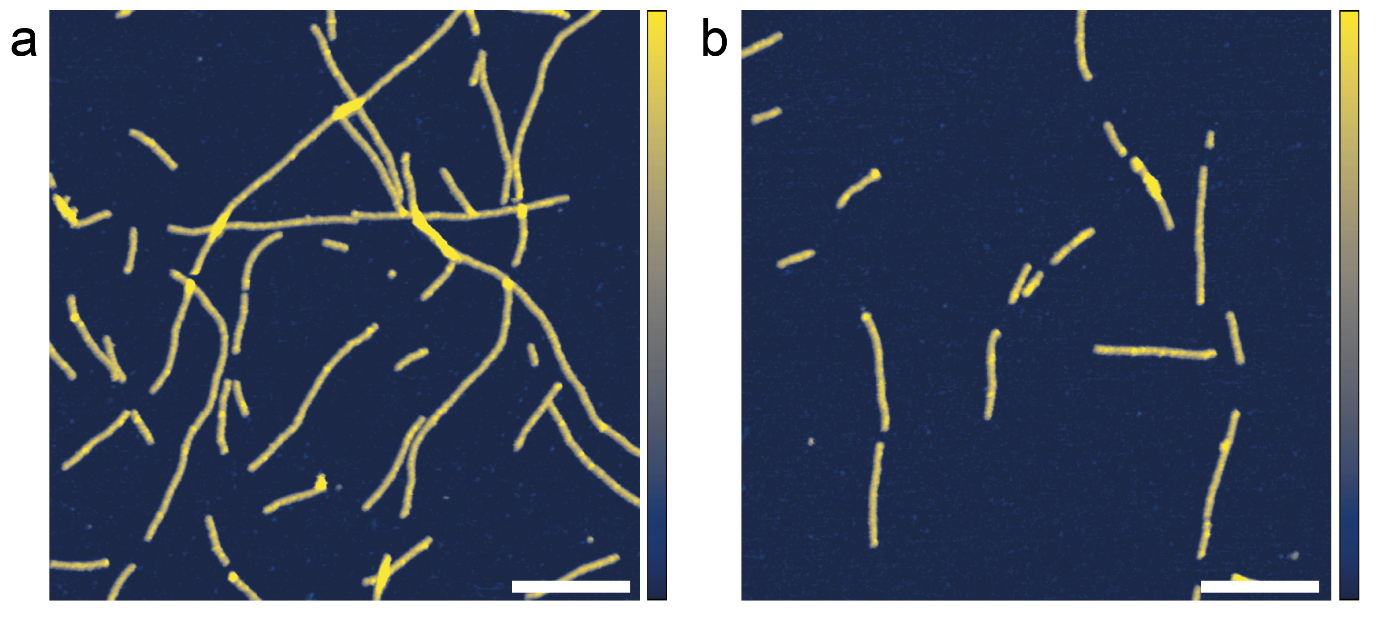


**Figure S2** PeakForce Tapping® AFM images of GMP solution dried on mica in a desiccator at room temperature, followed by thorough washing using MQ water. Imaging conducted in 100 mM KCl + 20 mM HEPES pH 7.4 solution. Scale bar is 100 nm and color z-scale is 0-4 nm for both images. (a) represents a high coverage region and (b) represents a low coverage region.


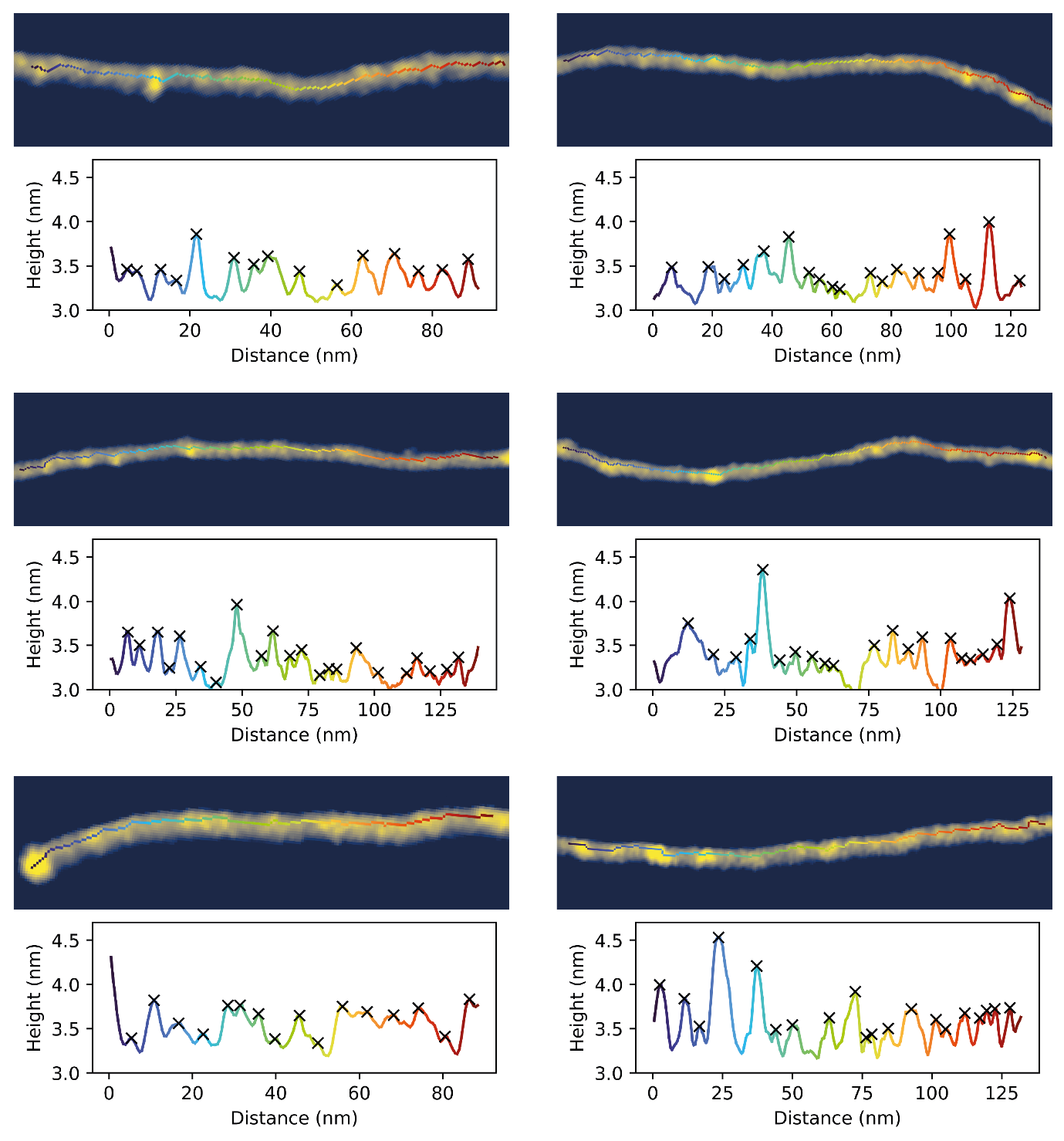


**Figure S3** Close-up images of single G-quadruplex segments from PeakForce Tapping® AFM of GMP solution dried on mica in a desiccator at room temperature, followed by thorough washing using MQ water. Imaging conducted in 100 mM KCl + 20 mM HEPES pH 7.4 aqueous solution. Color z-scales are 2-4 nm for all images. Skeletonised traces and corresponding height profiles with identified peaks are shown.


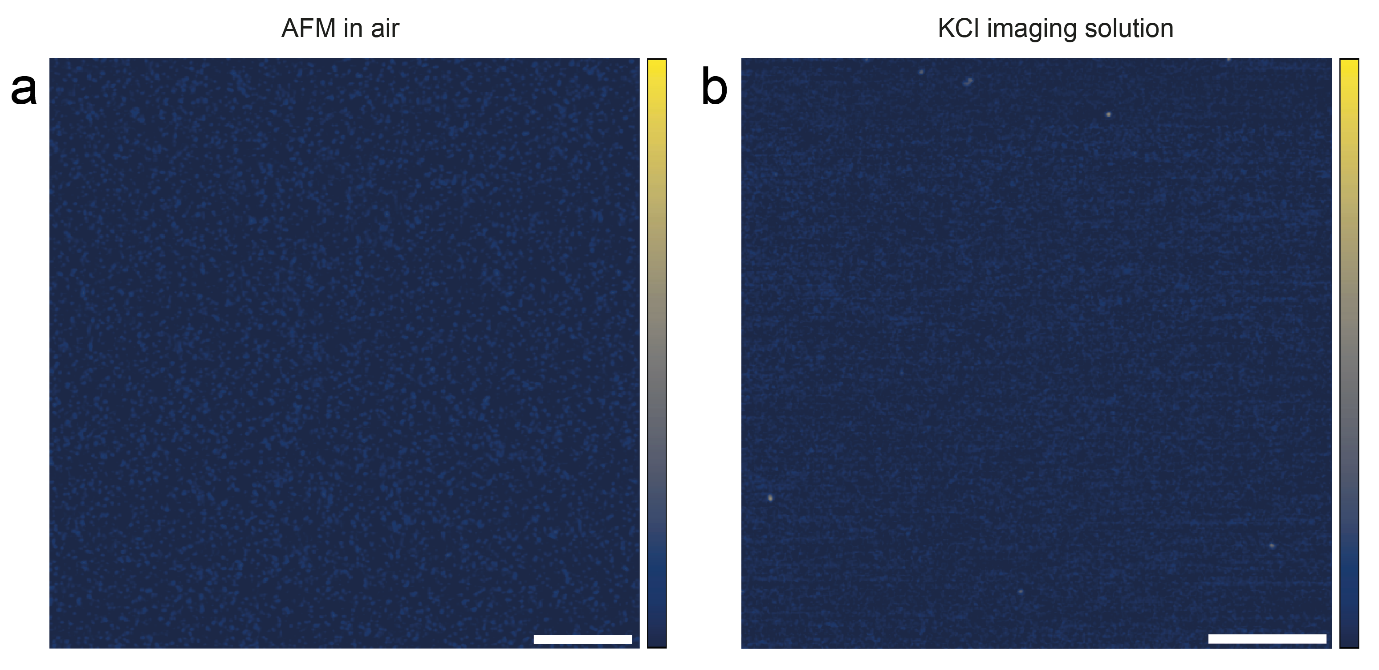


**Figure S4** Representative AFM images of 10 mM GMP solution incubated on mica surfaces. (a) GMP solution incubated for 5 min, followed by gentle rinsing using 1 ml MQ water and immediate drying using N_2_. AFM conducted in air. Scale bar is 500 nm. (b) GMP incubated in 25 mM MgCl_2_ + 10 mM TRIS pH 7.4 for 5 min, followed by gentle washing with and imaging in 100 mM KCl + 20 mM HEPES pH 7.4. Scale bar is 200 nm. Color z-scale is 0-3 nm for both images.


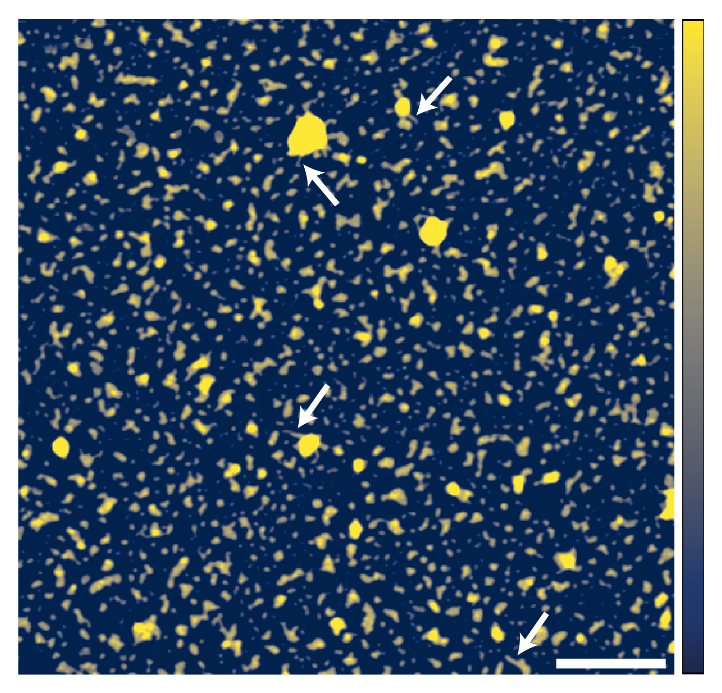


**Figure S5** Representative AFM image of 10 mM GMP solution dried on mica under N_2_ for 1 hour, followed by gentle rinsing using 1 ml MQ water and immediate drying using N_2_. AFM conducted in air. Arrows indicate short rod-like structures. Scale bar is 500 nm and color z-scale is 0-3 nm.


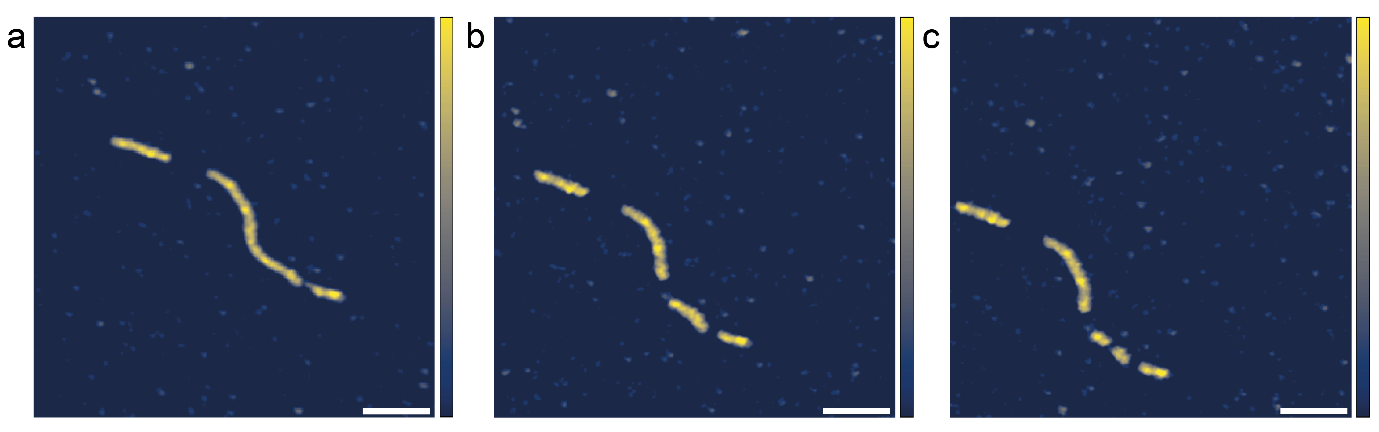


**Figure S6** PeakForce Tapping® AFM images of GMP solution dried on mica in a desiccator at room temperature, followed by thorough washing using MQ water. Imaging conducted in 3 mM NiCl2 + 20 mM HEPES pH 7.4 solution. (a-c) time series showing three AFM images at same location acquired sequentially. Scale bars are 50 nm and color z-scale is 0-4 nm for all images.


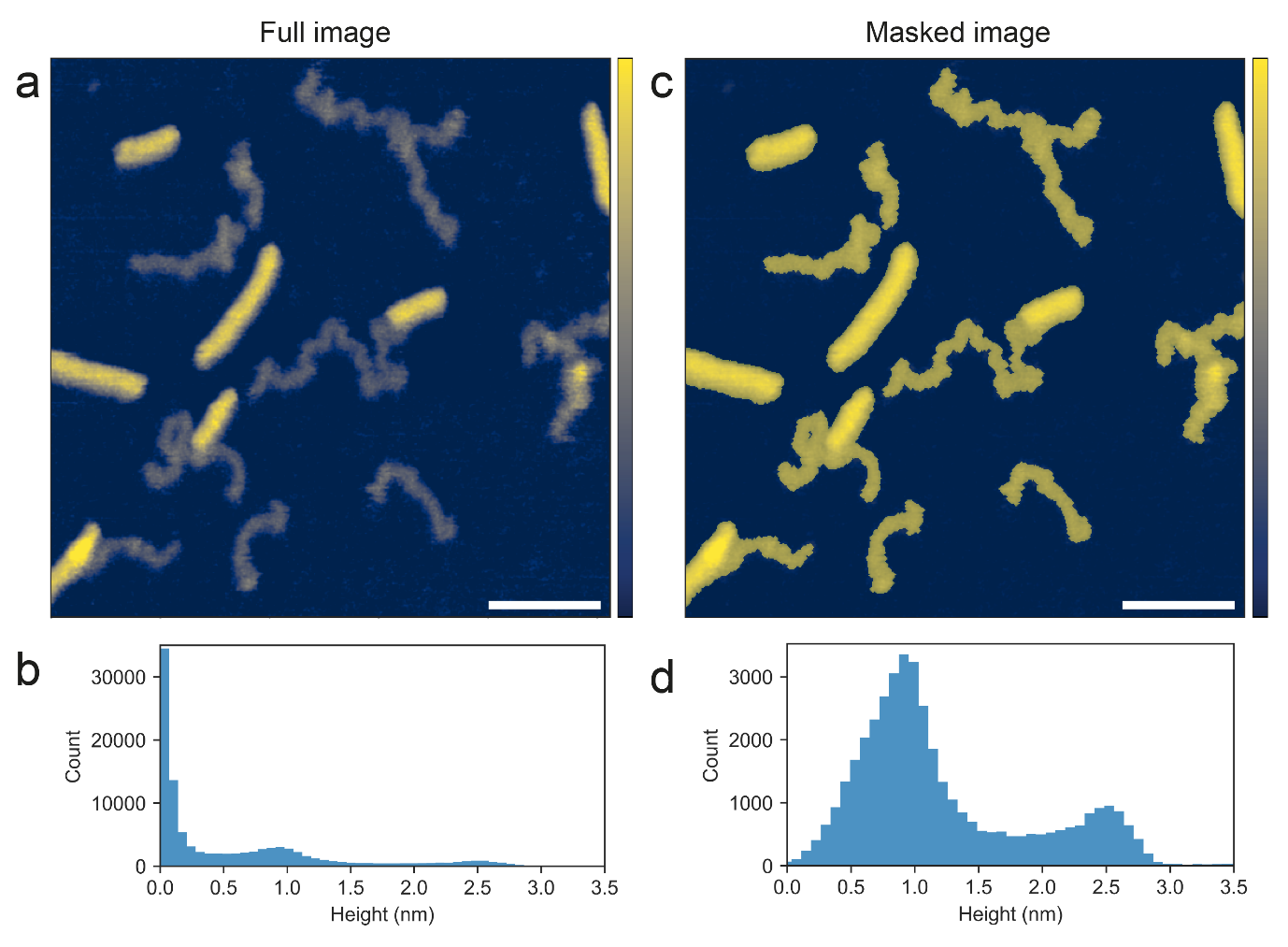


**Figure S7** PeakForce Tapping® AFM image of the products after hot (80°C) wet/dry cycles of the self-assembled G-quadruplexes. Imaging conducted in 100 mM KCl + 20 mM HEPES pH 7.4 aqueous solution. Scale bar is 50 nm and color z-scale is 0-3 nm. (a) Full AFM image and (b) corresponding height distribution of all pixels in the image. (c) Masked AFM image and (d) corresponding height distribution of the pixels under the mask.


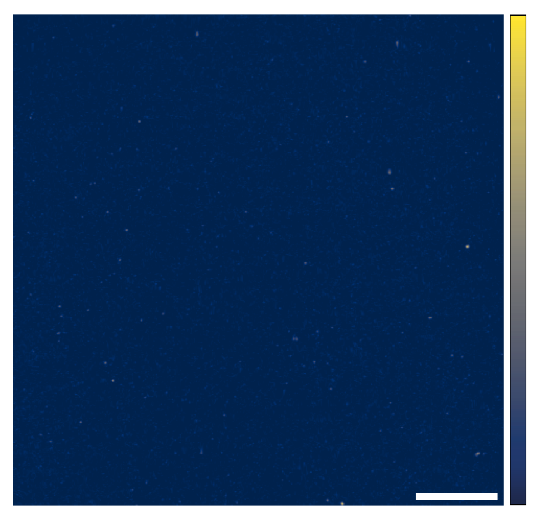


**Figure S8** Representative AFM image of a mica surface after hot (80°C) wet/dry cycles of solely MQ water, followed by gentle rinsing using 1 ml MQ water and immediate N_2_ drying. AFM conducted in air. Scale bar is 500 nm and color z-scale is 0-2 nm.


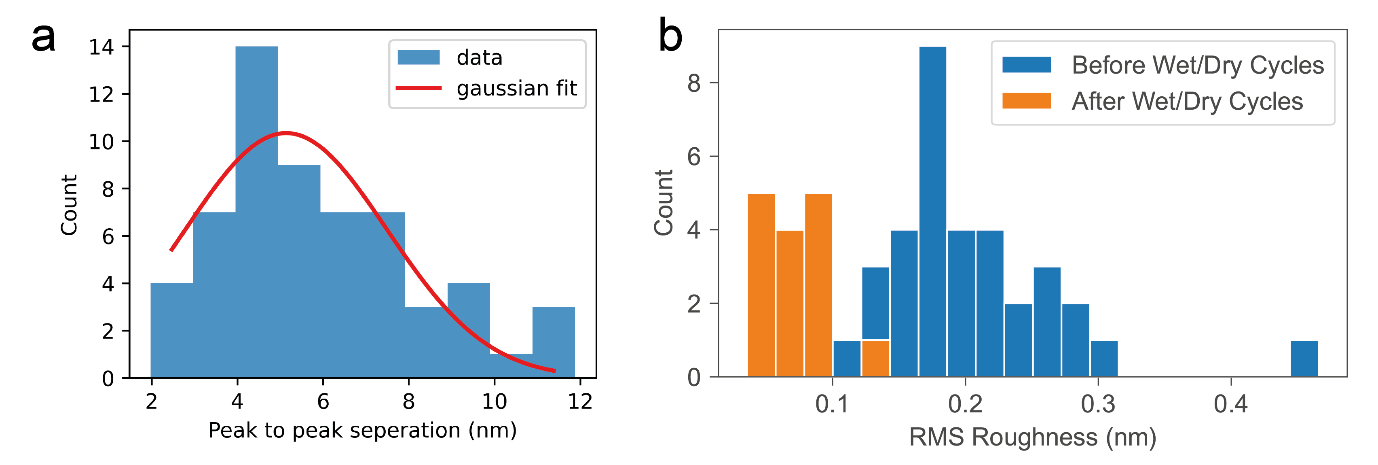


**Figure S9** (a) Histogram of peak-peak separations in height profiles from multiple G-quadruplexes after wet/dry cycles (N=15). Gaussian fit resulted in a mean peak-peak separation of 5.1 ± 2.4 nm. (b) Distribution of RMS roughness calculated for multiple G-quadruplex height profiles before (N=33) and after (N=15) wet/dry cycles. Average RMS roughness was calculated to 0.21 ± 0.07 (before wet/dry cycles) and 0.07 ± 0.02 (after wet/dry cycles).


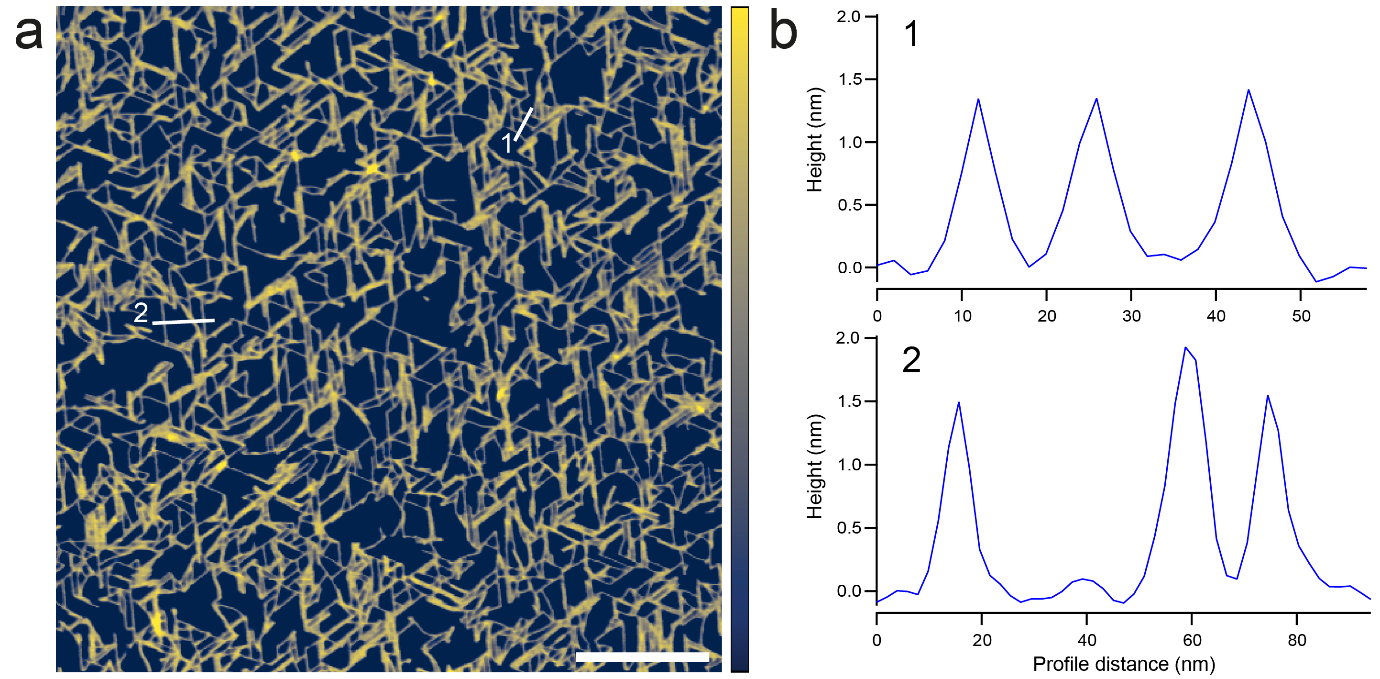


**Figure S10** (a) Representative AFM image after drying of 15 µM solution mixture containing GMP, CMP, UMP and AMP on mica in a desiccator at room temperature. AFM conducted in air, scale bar is 200 nm and color z-scale is 0-2 nm. (b) Height profiles corresponding to ‘1’ and ‘2’ in the AFM image.


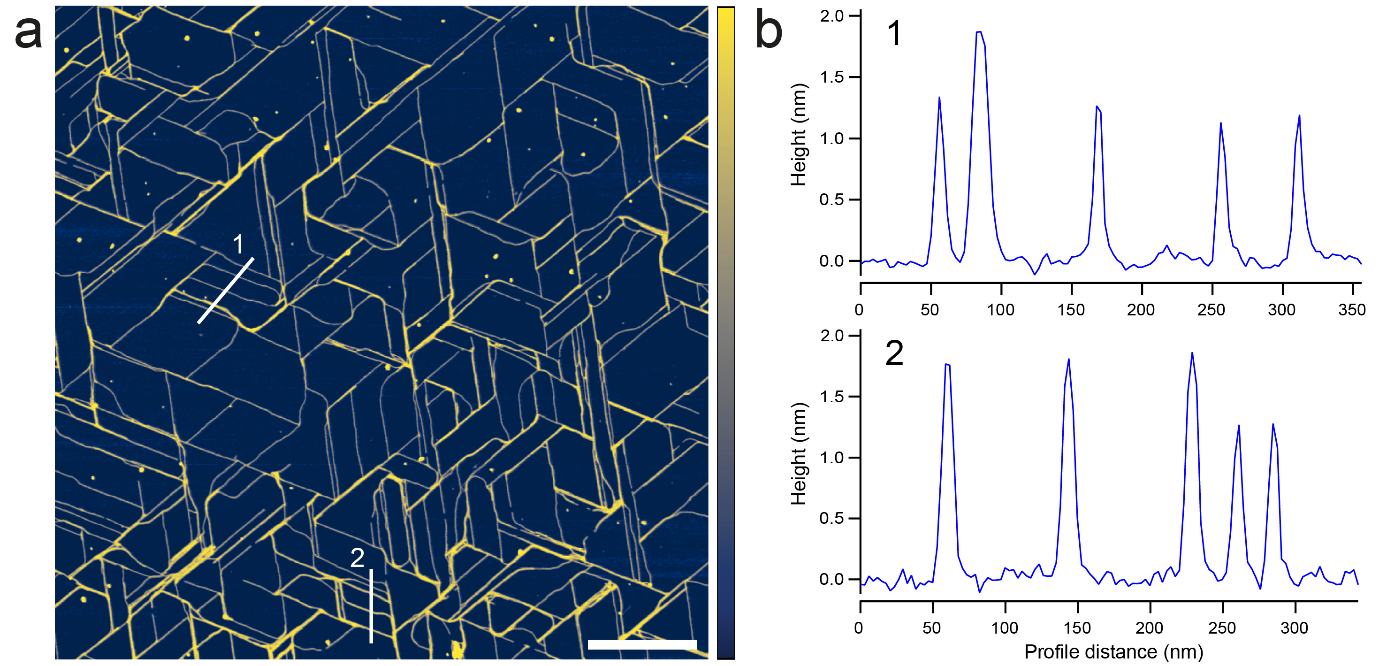


**Figure S11** (a) Representative AFM image of numerous filamentous structures after drying of 25 µM guanine solution (pH 6) on mica in a desiccator at room temperature. AFM conducted in air, scale bar is 500 nm and color z-scale is 0-2 nm. (b) Height profiles corresponding to ‘1’ and ‘2’ in the AFM image.

**Supplementary Methods**

The following supplementary methods were employed to resolve further structural or chemical details for the RNA-like structures produced by hot wet/dry cycling and visualized by AFM. However, no polymers could be detected. Given AFM evidence that the RNA-like structures contain covalently bonded GMP monomers, we attribute their lack of detection by bulk analytical chemistry techniques to the inability to solubilize and concentrate them to sufficient levels.

*Sample preparation*

After wet/dry cycling, we attempted to solubilize and extract the RNA-like structures from the mica substrates by incubating with ionic solutions or pure MQ water at 50 °C for 4 min, followed by collection into Eppendorf DNA LoBind tubes. Collected samples were filtered using ZipTip C18 pipette tips (Millipore) to retain and purify any polymers, which were subsequently eluted with 10 µL of 1:1 HPLC-grade acetonitrile/MQ water. These eluates were analyzed by bulk analytical chemistry techniques.

*Electrospray ionization mass spectrometry (ESI-MS)*

ESI-MS was performed on a SolariX XR mass spectrometer (Bruker) in negative mode. Samples were diluted 1:20 in 10 mM ammonium formate (pH ~7) and injected at 240 µl/h. Nebulizer pressure was 1 bar, and a capillary voltage of 3400 V was applied for ionization. Desolvation was achieved with drying gas at 200 °C (4 L/min). Data were acquired over 150–4000 m/z (300-2100 m/z focus region) with 3 s accumulation averaged over 32 scans.

*Capillary electrophoresis*

Capillary electrophoresis was performed on an Agilent 7100 CE system with diode-array detection (DAD) for UV/vis absorption. Separation was carried out using a 35 cm × 50 µm (internal diameter) fused silica capillary (Agilent) with 20 mM inorganic phosphate buffer (pH 6.2). Samples were diluted 1:5 in buffer and were injected for 20 s. Separation was conducted under a voltage of -20 kV at room temperature.

*Fluorescence Spectroscopy*

Fluorescence spectroscopy was performed on a Jasco FP-8350 spectrofluorometer using SYBR^TM^ gold (Thermo Scientific) as the fluorophore. Samples were mixed 1:1 with SYBR^TM^ gold (2X concentrate), placed in a 1.5 mm low-volume cuvette, and measured at room temperature with excitation at 480 nm and emission recorded from 490–750 nm.
